# Case of Large Incysted Tumour Connected with the Kidneys

**Published:** 1816-01

**Authors:** Edward Jackson


THE LONDON
### Medical and Physical Journal.

1 OF VOL. XXXV.] JANUARY, 1816. [NO. 203.

"For many fortunate discoveries in medicine, and for the detection of numerous errors, the world is indebted to the rapid circulation of Monthly Journals; and there never existed any work to which the Faculty in EUROPE and AMERICA were under deeper obligations than to the Medical and Physical Journal of London, now forming a long, but an invaluable, series."—RUSH.

*For the London Medical and Physical Journal.*

### *Case of large incysted Tumour connected with the Kidneys;* by EDWARD JACKSON, Esq.

J. B., a poor labouring man, fifty-six years of age, in the month of May last, complained of great pain in the belly, which came on suddenly as he was returning home after his day's work; his pulse at this time was quick, hard, and strong, and the tongue white and dry; twelve ounces of blood were immediately taken from the arm, and some purgative medicines, consisting of pills of extract of colocynth with calomel and a solution of sulphate of magnesia in mint-water, administered. The medicines operated very speedily, and on the following morning the pain had very much abated; in a few days, however, it returned, and recourse was had to the same means, which had been so useful in subduing the first attack, and with nearly the same result; the pain, however, though considerably lessened, was by no means entirely done away, and from this time the disease put on a more chronical appearance. A dull and heavy pain was constantly felt in the part first affected, and in the course of a short time a swelling was perceived on the left side of the abdomen, a little above the umbilicus, which was hard and tender to the touch; this kept gradually increasing till the patient's death, (a period of four months), at which time it was of an enormous size, its upper extremity touching the sternum, and its lower resting upon the left thigh. During the progress of this disease there was but little variation in the symptoms, the pain in the affected part was constant, though not very severe; the tumour gradually increased in size, and was hard and unyielding to the touch; the excretions were healthy in appearance, but the bowels were seldom or never relieved without the help of aperient medicines; the urine was rather small in quantity; the appetite moderately good; the tongue, which in the first instance had been dry and furred, now became moist and clean at the edges; the legs and feet had, at one time, been œdematous; but this symptom disappeared as soon as he took to his bed; as the disease advanced the appetite decreased, and eventually the stomach lost the power of retaining food, almost every thing being vomited up soon after it had been swallowed. For some weeks previous to his death he was supported by very minute quantities of mint-tea or weak spirit and water; the body of course became exceedingly emaciated.

The mode of treatment consisted of general and topical bleeding, blistering, aperient medicines, alteratives, with opiates, diuretics, and mercurial frictions; but it did not appear that any of these means had the slightest effect in checking the progress of the complaint, which kept gradually increasing till death put an end to the poor man's sufferings.

On inspecting the body a very large tumour was found, apparently occupying the whole of the abdominal cavity, in shape and appearance very much resembling a large bullock's heart, the veins on its surface being very similar to the coronary vessels of that organ; all the viscera of the abdomen were completely obscured by this tumour, with the exception of the colon, which lay obliquely on its surface and adhered very firmly to it; it became necessary, therefore, to empty the tumour of its contents, in order to ascertain its true nature and character. A small puncture being made in its most prominent part, a quantity of dark coloured fluid was discharged, which was at first thin and transparent, but presently became viscid and opaque; and eventually some pieces of a substance resembling spunge, were brought up in each basin full of the fluid. Of this fluid fifteen pints were collected and measured, and, reckoning what was lost on the tumour, being first opened, the quantity could not be less than two gallons; it was of a peculiar odour, somewhat resembling blood that has been a few days extravasated, and had an unctuous and rather an adhesive feel. On examining the parts, it was immediately evident that the seat of disease was in the kidney, and that the sac was composed of the proper membrane of that gland covered by peritoneum, to which it firmly adhered, forming a compact and strong bag about one-eighth of an inch in thickness.

The ureter was found entering the sac at its lower and posterior part, but was unfortunately detached in attempting to raise the tumour from its connection with the loins. It appeared of the ordinary size, and healthy in its whole course, course, but the extremity next the kidney was plugged up by a firm coagulum of blood.

The interior of the sac was smooth, except at the bottom, where it was partly covered by the loose spungy substance before mentioned, which was easily separated by the fingers; the opposite kidney was of the usual size, and the rest of the viscera perfectly healthy, though the liver appeared smaller and of a paler colour than usual.

Want of time would not permit me to make a more minute examination, and it is to be lamented that the task of investigating and describing this extraordinary case did not fall to abler hands.

I have since learnt that a few weeks previous to the commencement of this poor man's illness, he received a severe blow on the loins by the handle of a pick-axe, but it did not appear to be productive of much inconvenience at the time, and it is worthy of remark, that in the whole course of his illness he had not any symptom which could lead me to suspect the kidney to be the seat of disease.

*Guildford;*

*November* 10, 1815.

---

*For the London Medical and Physical Journal.*

